# Volatile Profile and Physico-Chemical Analysis of *Acacia* Honey for Geographical Origin and Nutritional Value Determination

**DOI:** 10.3390/foods8100445

**Published:** 2019-09-27

**Authors:** Niculina M. Mădaş, Liviu A. Mărghitaş, Daniel S. Dezmirean, Victorita Bonta, Otilia Bobiş, Marie-Laure Fauconnier, Frédéric Francis, Eric Haubruge, Kim B. Nguyen

**Affiliations:** 1 Department of Apiculture and Sericulture, University of Agricultural Sciences and Veterinary Medicine, Mănăştur st, 3-5, 400372 Cluj-Napoca, Romania; mmadas@gmail.com (N.M.M.); lmarghitas@usamvcluj.ro (L.A.M.); ddezmirean@usamvcluj.ro (D.S.D.); 2 Department of Functional and Evolutionary Entomology, University of Liège, Gembloux Agro-Bio Tech, Passage des Déportés, 2, 5030 Gembloux, Belgium; frederic.francis@ulg.ac.be (F.F.); e.haubruge@ulg.ac.be (E.H.); kim.nguyen@beeodiversity.com (K.B.N.); 3 Life Science Institute, University of Agricultural Sciences and Veterinary Medicine Cluj-Napoca, Manastur st. 3-5, 400372 Cluj-Napoca, Romania; victorita.bonta@usamvcluj.ro; 4 Laboratory of Chemistry of Natural Molecules, University of Liège, Gembloux Agro-Bio Tech, Passage des Déportés, 2, 5030 Gembloux, Belgium; marie-laure.fauconnier@uliege.be

**Keywords:** acacia honey, gas-chromatography, HPLC, sugar spectrum, volatile organic compounds

## Abstract

Honey composition and color depend greatly on the botanical and geographical origin. Water content, water activity and color of 50 declared acacia samples, collected from three different geographical zones of Romania, together with chromatographic determination of sugar spectrum were analyzed. A number of 79 volatile compounds from the classes of: Alcohols, aldehydes, esters, ketones, sulphur compounds, aliphatic hydrocarbons, nitrogen compounds, carboxylic acids, aromatic acids and ethers were identified by solid-phase micro-extraction and gas-chromatography mass spectrometry. The overall volatile profile and sugar spectrum of the investigated honey samples allow the differentiation of geographical origin for the acacia honey samples subjected to analysis. The statistical models of the chromatic determination, physicochemical parameters and volatile profile was optimal to characterize the honey samples and group them into three geographical origins, even they belong to the same botanical origin.

## 1. Introduction

Nutritional value of honey [1] is due to the chemical composition of simple sugars and all the substances (vitamins, enzymes, minerals, free amino-acids, phenolic and volatile organic compounds—VOCs), that have been described in many studies made over the time [2,3,4,5].

The bioactive properties of honey are related to the botanical and geographical origin, being influenced mainly by the plants from where the bees collect the nectar, and thus by the place where these plants grow [2,5,6,7,8,9]. When talking about botanical origin, honey may be classified as floral (derived from nectar of melliferous plants) or non-floral (honeydew) (derived from sweet substances released by aphids on different plant parts which bees collect and transform similarly to floral nectar). Botanical and geographical authentication of honey is a market feature, and for this reason different research groups and specialized laboratories have established a list of methods and parameters to be determined, for the correct classification of honey samples.

Monofloral honey is characterized by a required percentage of specific pollen, different in every national standard, ranging between 10 and 20% (orange honey) to 70–90% for eucalyptus honey [10,11,12,13]. Establishing the monoflorality of honey have also economic importance, because specific honey, with nectar and pollen belonging predominantly to a certain species, may have higher selling price and beekeepers have higher incomes obtained from its marketing.

However, only pollen analysis may cause classification problems, due to the fact that certain plants are underrepresented in pollen (lavender, citrus) and additional analysis are needed to complement the uniflorality decision. These analysis may be physico-chemical [14,15], spectrophotometric and chromatographic techniques for possible markers determination (sugars, phenols or volatiles) [9,16,17,18,19,20,21,22].

Honey aroma is given by volatile organic compounds coming from plant nectar, developed through different biosynthetic pathways, thus is closely related to botanical origin [20]. Additionally, some of the flavor constituents may come from the bees in the enzymatic process of transforming the nectar into honey [20].

Different classes of volatile compounds are reported in honey, the main classes being: furans, alcohols, aldehydes, ketones, terpenes, nor-isoprenoids, acids and pyrene derivatives [9,19,20,22].

Having different landforms and a very rich flora, Romania have the possibility to produce many types of honey, from monofloral (acacia, linden, rape, sunflower, raspberry, heather, mint, etc.), to multifloral (mountain, meadow) and last but not least honeydew honey [5,14,16,21,23,24,25,26].

Romanian honey volatiles were studied until now [21,27,28], but the comparative analysis of volatiles of the same type of declared honey, from different geographical origins of Romania, was not made until now. The main purpose of the present study was to assess the volatile composition on the creation of aroma present in acacia samples from different geographical regions of Romania, using solid phase micro-extraction; gas chromatography, mass spectrometry (SPME/GC/MS) technique.

## 2. Materials and Methods

Most of the physico-chemical methods used in honey analysis are mainly used for quality control, but some of them, like electrical conductivity and sugar spectrum determination, may be used for botanical or geographical origin determination.

### 2.1. Honey Samples

**Figure 1:**
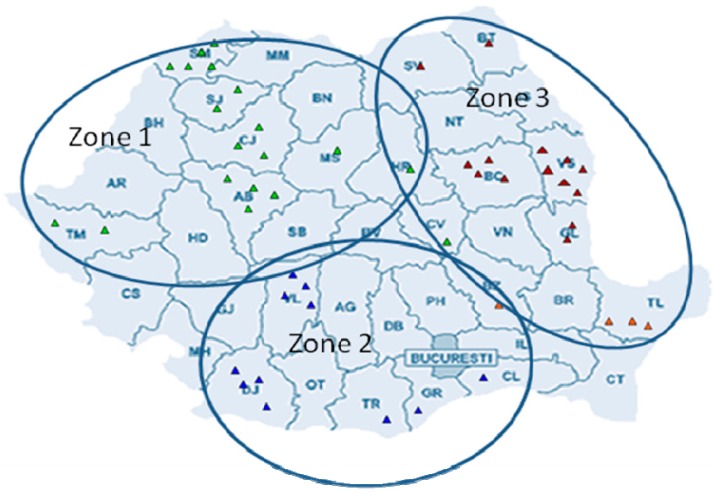
Geographic origin of investigated acacia honey samples (Green triangle – Zone 1; Blue triangle – Zone 2; Red triangle – Zone 3 of sample harvesting).

Based on the main objective of this study, the strategy was to collect honey samples from different meliferous areas, in order to study the typicity of *Robinia pseudoacacia* honey from Romania. Fifty honey samples were collected directly from beekeepers; the selection was made in three distinct geographic regions with different pedological characteristics (diverse landforms, proportionally distributed and concentrically arranged around Transylvania Plateau), and climate (temperate-continental, with regional climatic variation due to the variated topography), with predominant *Robinia pseudoacacia* trees as nectar sources. Zone 1 was represented by Transilvania region (Alba, Cluj, Covasna, Harghita, Mures and Satu Mare counties), Zone 2 was the southern part of Romania (Călăraşi, Dolj, Giurgiu, Teleorman and Vȃlcea counties) and Zone 3 were mainly eastern part of Romania (Bacău, Botoşani, Buzău, Galaţi, Suceava, Tulcea and Vaslui counties) (Figure 1). Samples were kept in glass jars, at 4 °C, in dark places until analysis.

### 2.2. Chemicals and Reagents

All chemicals and reagents were analytically grade purity. Ultrapure water was made with MilliQ Integral SG Wasseraufbreitung (Darmstadt, Germany). Sigma Aldrich (Steinheim, Germany) provided fructose, glucose, sucrose, maltose, isomaltose, trehalose and erlose. A mixture of homologues n-alkanes (C_7_–C_30_) (Sigma, Darmstadt, Germany) was used for gas chromatographic determinations of VOCs.

### 2.3. Physico-Chemical Analysis

Selective physicochemical parameters were determined according to Romanian standard [29] and Harmonized Methods of International Honey Commission [30]. Water content was determined refractometrically (Abbe digital refractometer, WYA-S, Selecta, km. 585, ABRERA Barcelona, Spain) Spain). The respective content was expressed as mg/100 g; the water activity was measured using a water activity meter Decagon, AquaLab CX-3 (Pullman, WA, USA). Measuring cells were placed minimul 2 h into a thermostatated chambre “Zanotti” type at 20 °C and measured afterwards. Equipment etalonation was made by measuring distilled water activity (a_w_ = 1.000 ± 0.003 La 20 °C) and a standard of lithium chloride (a_w_ 0.120 ± 0.003 La 20 °C). Color has been determined using refractrometric method in uniform color space CIELAB (Supplementary Figure S1), where any color may be specified by using rectangular coordinates L*, a*, b*. Axes a* and b* represent color tonality, and L* axis represent the luminosity.

Measurement principle is placing the sample in a three-dimensional space, in a position in three-axis rapping as follows:

L* which place the sample on the axis that has black and white;
a* which place the sample on the axis that has green and red;
b* which place the sample on the axis that has blue and green.

Measuring equipment, HUNTERLAB miniscan XE (Reston, VA, USA), measures by spectrophotometry the reflectance of the sample color.

L* value indicate the degree of brightness, positive values of a* indicates red zone, negative values of a*, green component; positive values of b* indicates yellow zone and negative values of b* indicates blue component.

The HPLC analysis of the carbohydrates is carried out on a modified Alltima Amino 100Å stainless steel column (Hicrom, Berkshire, UK) (4.6 mm diameter, 250 mm length, particle size 5 μm) following IHC method modified in APHIS-DIA Laboratory [16]. The SHIMADZU instrument (LC–10AD VP model, Shimadzu, Kyoto, Japan) was equipped with degasser, two pumps, autosampler, thermostat oven, controller and refractive index detector. The injection volume was 10 μL and the flow rate 1.3 mL/min. The mobile phase is a solution of HPLC purity acetonitrile and ultrapure water (80/20 *v/v*). Briefly, 5 g of honey were dissolved in water (40 mL) and transferred quantitatively into a 50 mL volumetric flask, containing 25 mL methanol HPLC grade purity and filled up to the mark with water. The solution was filtered through a 0.45 µm membrane filter, collected in sample vials and placed in autosampler for analysis. For the quantification of main sugars, different calibration curves in the range 50–0.25 g/100 g (fructose 50–20 g/100 g; glucose 10-40 g/100 g; sucrose 0.3–15 g/100 g; turanose, maltose, isomaltose, erlose 0.25–5 g/100 g), with regression coefficients (*R*^2^) higher of 0.998 were obtained. The results are expressed in g/100 g honey.

### 2.4. Solid-Phase Microextraction and GC-MS Analysis

For the evaluation of the VOC profile in honey, a SPME automatic support was used, equipped with a fiber type 50/30 divinylbenzene/carboxen/polydimethylsiloxane (DVB/CAR/PDMS) (Supelco, Bellefonte, PA, USA). Thus, 4 g of each honey sample in a 30 mL headspace vial, sealed afterwards with a screwed cap with PTFE/silicon septa. Samples were homogenized and heated to 40 °C for 30 min. After equilibration, the fiber was introduced into the vial headspace through the septum and exposed to the sample for 30 min.

Volatile analyses were performed using a GC Agilent 7890A system (Wilmington, DE, USA) with injectors both with and without mobile phase flux dividers and an AGILENT 5675C inert XLEO/CI MSD mass spectrometry with a triple-axis detector. The SPME fiber was desorbed at 250 °C for 5 min without dividing of the mobile phase. The separation of VOC was performed on a Hewlett-Packard SP-5-DBWAX (30 m × 0.25 mm i.d. and 0.25 µm film thickness) (Bellefonte, PA, USA).

The column temperature was maintained in the column oven following the gradient: the initial temperature of 35 °C was kept constant for 5 min, raised with 3 °C/min until it reached 50 °C. After this, it raised with 5 °C/min until 180 °C, with 6 °C/min up to 250 °C and held at this value for 10 min. The temperatures of the injector and detector were set at 250 °C and 230 °C, respectively. Helium was used as carrier gas (>90% purity).

### 2.5. Data Analysis

The identification of the compounds was made by retention time and by comparing the mass spectra obtained with standard mass spectra from the “Pal 600” spectra library. Also, the retention indices (RI) were calculated by injecting a mixture of homologues n-alkanes (C7–C30) under the same chromatographic conditions (Sigma, Darmstadt, Germany) [31]. Furthermore, the indices calculated for the selected compounds were compared with the literature data (www.pherobase.com). For a better quantification of the results, the method of normalization area was used, each compound’s concentration being calculated according to the following expression:

X% = (A_X_/ƩA_i_) (1)

where: A_x_: The compounds surface, ƩA_i_: The sum of all identified compound’s surface.

### 2.6. Statistical Analysis

Physico-chemical analysis were performed in triplicate, data being expressed as mean ± SD. Differences between means were determined using one-way ANOVA test (IBM SPSS Statistics for Windows, Version 25.0, IBM Corp., Armonk, NY, USA). Variance analysis, ANOVA, calculates the ratio between the variation caused by intergroup and intragroup differences and establishes whether this ratio is large enough in order to distinguish the groups.

## 3. Results and Discussion

### 3.1. Physicochemical Parameter Values for Acacia Honeys

Acacia honey in general has a low water content [14,32,33,34,35], which can be observed also in this study. The values obtained are within the limits pertaining to the honeys found in the temperate climate, especially in Europe.

In order to carry out a primary interpretation of the data obtained for this parameter and to have a clear image of its variability in accordance with the geographical origin, a box plot graph of these values was constructed for each zone. The box plot diagram summarizes the charts for the five values specific to their spread in each zone (the minimum, the first quartile, the median, the third quartile and the maximum) and the extreme values. In this chart, one can observe a central line for each box plot, representing the median of the values measured for each zone. If the median is closer to the lower margin, the value spread leans towards the left, and if it is closer to the higher margin, the spread leans towards the right. For Zone 1, the maximum of the median was found to be 16.704, for Zone 2, 17.416, and for zone 3, the median value was 17.088. The box plot shows 50% of the values, and its size shows result variability. The horizontal lines above and below the box are traced from between the minimum and the maximum for each zone, so that the minimum values are 15.4, 15.8 and 17.1 for zones 1, 2, 3, respectively. The extreme values are placed outside the box plot and are marked with “*”, accompanied by the sample number. In the case of water content determination, two extreme values have been found, samples S15 (belonging to Zone 1) and S25 (Zone 2) (Supplementary Figure S2).

The values obtained for water activity ranged between 0.540 (S51, Zone 1) and 0.690 (S25, Zone 2) (Table 1).

A water activity value of 0.562 was recorded for Slovakian acacia honey [36], 0.490 for Czech honey [33] and 0.81 for Romanian honey [37].

The L* value shows the brightness level, positive values of the parameter a* show red zone, negative values for a* show the green component, positive values for b* show the yellow zone and negative values for b* indicate the blue component. The values for L* (Table 2) were between 50.19 and 62.70, and the average obtained after measuring all the samples was 58.96. These values are in accordance with those measured by Bertoncelj et al. [38] for Slovenian honey (64.4), some Spanish honeys (51.62–56.11) [38] but are much higher than those found for Slovakian honey (11.29) [36] and other Romanian acacia honeys (48.96) [37].

The value for parameter a* (Table 2) lies between –1.28 and 5.80. The average obtained by the measuring of all the samples was 0.08 ± 1.12. A wide distribution can be observed when determining this parameter, as samples were situated in the green zone (negative a*), as well as in the red zone (positive a*). Only positive values were found for this parameter by Kasperová et al. (4.07) [35] in the case of Slovakian honey, Romanian honey [37], whereas Bertoncelj et al. [38] found only negative values for Slovenian acacia honey (−2.82), as well as Karabagias et al. [8] for Portuguese honey. As it can be observed in Table 2, the positive and negative values obtained for this parameter cannot be linked to geographical origin or with the sample year of harvest. Each zone has shown both negative and positive values. The same characteristic presented Spanish honey [39].

As for b* parameter values (Table 2), the recorded minimum was 6.23, and the maximum 30.41. The measurement average for all zones was 15.70. Despite the fact that the zone between the minimum and maximum values of the b* parameter is relatively vast, the average obtained for acacia honey in correlated to the value found for Slovenian acacia honey [38] (17.95) and to that of Slovakian honey (14.66) [36], but much higher than other Romanian acacia honeys [37].

The highest median value for the L* parameter was recorded in the case of the samples from Zone 1 (59.315), a value close to the one from Zone 3 (59.062), while the value for Zone 2 has shown the lowest value (58.177). Variance values are higher than one and show a greater degree of variation. The results of the ANOVA *p* = 0.5662 and *F* = 0.575 test show that there are no significant differences between samples when measuring this parameter. Thus, the samples are homogenous (Table 3).

**Figure 2:**
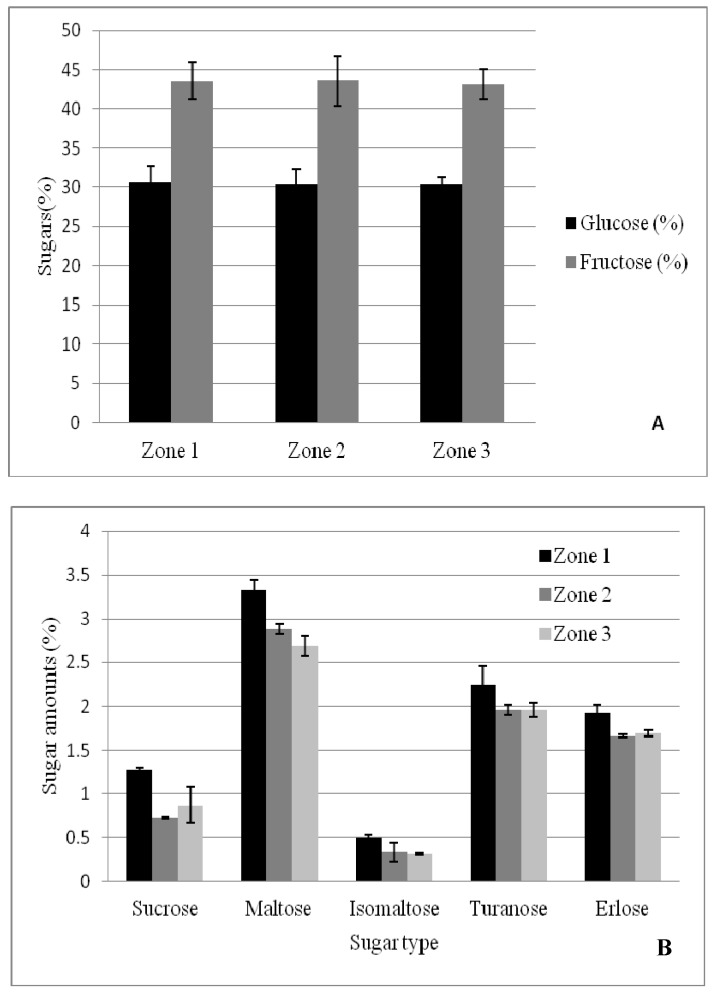
Glucose and fructose content (**A**) and minor sugars (**B**) of the analyzed samples (mean value of three repetitions ± SD).

The results obtained for each sugar are presented in Figure 2A, B. Each identified and quantified sugar was individually discussed, as the values obtained are extremely important to determine and authenticate the botanical origin of the honey samples.

As far as the glucose content is concerned, the values obtained for the 50 analyzed samples ranged between 22.32% and 43.71%. The mean value obtained for every zone was: 30.57 (Zone 1), 30.40 (Zone 2) and 30.37% (Zone 3).

Generally, acacia honey has an average glucose content of 26.3% [40]. Romanian acacia honey has values ranging from 30.87% [41] and 32.58% [14]. The results obtained by analyzing these samples fell within these limits and proved to be very similar to other acacia honeys in Europe [42,43].

The fructose content is a very important parameter in analyzing acacia honey. The fluid state of this type of honey is due to a high fructose content and implicitly to a higher than 1.2 fructose/glucose ratio. The results obtained following the HPLC analysis of fructose in the honey samples (Figure 2A) ranged between 33.80% and 48.16%. The mean value of the fructose content for each zone was: 43.55 (Zone 1), 43.58 (Zone 2) and 43.15% (Zone 3). While determining this parameter, we could observe the fact that only 5 samples out of 50 had a lower than 40% fructose content. Similar values were obtained in the case of other acacia honey samples from Romania [14,41] or other acacia honey samples in Europe [42,43]. As it has already been mentioned, the fructose/glucose ratio is very important in acacia honey analysis. Sabatini et al. [40] found about a 1.66 ± 0.14 ratio in the case of acacia honey. The results obtained following the HPLC analysis of individual carbohydrates in the analyzed samples highlight (with three exceptions) the supraunitary values of this ratio, while 50% of the samples showed a > 1.5 ratio.

Sucrose is a very important indicator of honey authenticity, as standards require a maximum of 5% in authentic honey. The samples analyzed have significantly lower values, under no suspicion of falsification. The values registered ranged between 0.0 and 5.51%, with an average value of 1.0% (mean of all zones) (Figure 2B). Only one sample had a concentration higher than 5% (sample S58, Zone 1). Some of the analyzed samples had a sucrose content that proved to be under the detection limit of the method and device (0.02%). The results obtained in this study confirmed earlier studies [41,44,45,46,47].

The ANOVA analysis (the ratio between the variance produced by differences inside the groups and the variance produced by differences outside the group) shows that there are no statistic differences (*p* = 0.3010 > 0.05 and *F* = 1.23174 < *F*_crit_) between the samples harvested from the three production zones. 

Maltose is an important disaccharide found in honey with determined values ranging between 1.3% [40], 1.9% [48] and 2.5% [49] or a medium value of 3.53% obtained by Mărghitaş et al. [14] for acacia honey in Romania (Transylvania). It is generally found in nectar honey and particularly in acacia honey.

The values obtained after analyzing the samples in this study varied between 0.68 and 4.16%. The ANOVA variance analysis shows that the value of p is lower than 0.05 (*p* = 0.012177), but *F* is bigger than *F*_crit_. (*F* = 4.848788 and *F*_crit_ = 3.195056). Consequently, the hypothesis fails because there are significant differences between the samples.

Isomaltose is a disaccharide found in a relatively small quantity in honey, generally less than 1%. Depending on the carbohydrate determination method chosen (HPLC, GC or FTIR), but also on the botanical origin of honey, the quantity may differ, but remains low. The values obtained in the case of acacia honey varied between 0.14 and 0.79% and the mean value of the 50 samples was 0.39%. Similar values were obtained for the acacia honey in Italy [40], other acacia honeys in Romania [14] or the honey in Poland [49]. However, it was easy to observe that this disaccharide presented values two times higher for the samples collected in geographical Zone 1 (Figure 2B). The two other zones presented similar values, although geographically they are very different (Zone 2 being located on the south-west of the country, while Zone 3 represents the eastern part of the country).

Turanose is a sugar found in small quantities in honey. The use of high performance liquid chromatography, gas chromatography or nuclear magnetic resonance, made it possible for this disaccharide to be found by different researchers in small quantities in Portuguese heather honey [50], in Polish nectar honey [49], in 5 types of Italian honey [51], in Hungarian acacia honey [52] and in Turkish nectar and honeydew honey [53].

Turanose was quantified between 0.38 and 2.77%, with an average of 2.08% in the acacia honey samples used in this study. These values are generally higher than the ones found by the aforementioned authors who have analyzed acacia honey, but similar to other honey types analyzed. 

Erlose is a trisaccharide found in honey in different quantities, in accordance with its botanical origin. The studies conducted at the APHIS Laboratory (USAMV Cluj-Napoca) have shown a rather high concentration of erlose in acacia honey, compared to other honey types [41].

Although Goldschmidt and Burckert [54] highlighted erlose as a honey component since 1955, many researchers did not report this trisaccharide when using high performance liquid chromatography as a determination method [49,52,55]. The values obtained for the analyzed honey samples ranged between 0.09 and 3.19%.

The glucidic spectrum analyzed by refractive index high performance liquid chromatography has shown the presence of seven saccharides specific to acacia honey. The samples fall between the limits established for this honey type, due to the high fructose content, >1.2 fructose/glucose (F/G) ratio, small sucrose content and higher than one maltose quantities [40,42,43].

**Figure 3:**
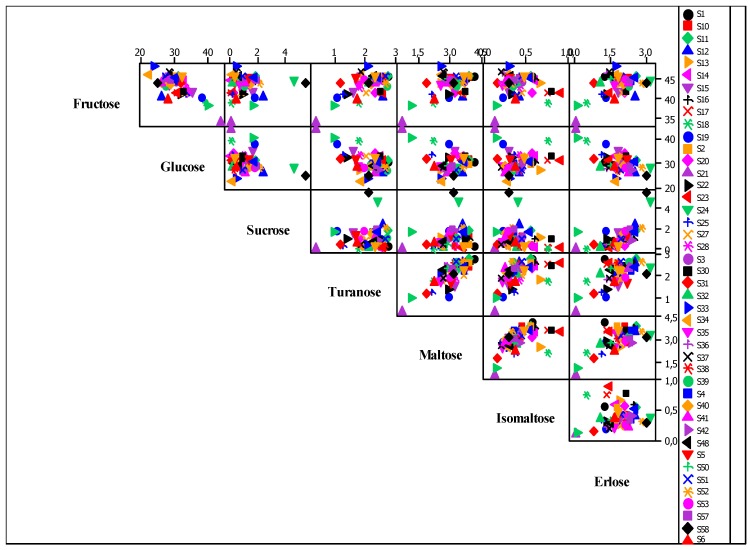
Graphic representation matrix plot of sugars.

In conclusion, the glucidic spectrum for acacia honey was established, and carbohydrate concentrations, especially fructose and F/G ratio, which show high values for this honey type. In order to illustrate the correlations between carbohydrates and to determine which samples are outliers, a graphical representation was made for all carbohydrates, called a matrix plot (Figure 3). 

Due to the vast database, high sample and analysis number, it is fit to use a Pearson correlation matrix in order to identify the correlation indices. These indices have a value ranging between (–1 to 1). Table 4, Table 5 and Table 6 show positive values, which means that there is a positive association of parameters, while negative values show an inverse dependence of the two parameters. The values colored in blue are significant for the 0.01 level, while the ones in yellow are significant of the 0.05 level.

For Zone 1, the best correlation was established between ash content and color. Also, positive correlations were found between different sugars, 0.72 for turanose–maltose and 0.79 for saccharose–erlose. The most significant negative correlation found for Zone 1 was between water activity–color (−0.841).

For Zone 2, significant positive correlations were found between sugars, 0.93 for turanose–maltose and 0.90 for sucrose–erlose. The most significant negative correlation was identified between the color parameters L*–a* (−0.94).

For Zone 3, the best correlation was established between water content and water activity, 0.92. The negative correlation found for Zone 3 was between color parameters (−0.80). 

### 3.2. Volatile Compounds

The GC-MS analysis method is a combination of two analytical techniques: capillary column GC, which separates the components of a mixture and mass spectrometry (MS), which supplies the information necessary in the structural determination of each constituent. The results obtained following the SPME/GC-MS analysis is shown as chromatograms (Supplementary Figure S3). Their interpretation is achieved by identifying mass spectrum and the retention time. These two parameters significantly contribute to the identification of each volatile compound found in honey. Also, retention time has a determining role in many cases where the constituents show identical mass spectra. Following the SPME/GC-MS analysis, 79 constituents were identified the honey samples: 72 compounds samples from Transylvania (Zone 1), 57 compounds in samples from Southern Romania (Zone 2) and 66 compounds in samples from Eastern Romania (Zone 3). From all identified compounds, only 54 are common to all 50 samples. The chemotype changes from one region to another, and the difference is constituted by the 6 compounds found only in one region and 9 compounds in two of the regions.

The interpretation of the chromatograms is achieved using the “Data Analysis” program. The noise level is set to 50 for the x-coordinate and the program goes over the chromatogram signal by signal according to this ratio. Identification of the compounds is achieved by comparing standard mass spectra from the “Pal 600” spectra library. The surface is calculated following the “apexing mass” model, which means measuring the surfaces in its entire mass. 

The next stage in the qualitative analysis is the calculation of the Kovats indexes. We have used this retention index in order to eliminate the relativity of the retention parameters by inserting some reference points in order to compare the retention times of chromatographic separation for each volatile compound found in honey. In this respect, a mixture of homologues n-alkanes (C_7_–C_30_) was used under the same chromatographic conditions (Sigma, Darmstadt, Germany), whose retention indices showed values between 600 and 2200. The retention indices specific to each compound is calculated as follows:

RI = 100 ((log tr_i_ − log tr*_n_*)/(log tr*_n_*_+1_ − log tr*_n_*)), (2)

where: RI: compound retention index, tr_i_: compound retention time, tr*_n_*: retention time of the adjacent and anterior alkane, tr*_n_*_+1_: retention time of the adjacent and subsequent alkane.

The expression of the retention index uses two reference points: the alkane with the same number of carbon atoms in its molecule (*n*) as the volatile compound identified as i, which will elute before the compound, and the second reference will be the alkane with *n*+1 carbon atoms in its molecule, which will elute after the i compound. Table 7 comparatively shows the retention indices calculated RI^b^ and the retention indexes found in literature. RI^a^ corresponds to each volatile compound found and the type of column used (polar column). The main bibliographical source used was the PHEROBASE database, which is one of the most comprehensive and up-to-date. 

The main volatile compounds of acacia honey identified undoubtedly are: 3-methyl-3-buten-1-ol for Zone 1, ethanol, acetic acid, 5-ethenyldihydro-5-furanone for Zone 2, acetone, 3-methyl-3-buten-1-ol, *trans*-linalool oxide, benzemethanol for Zone 3. Among the secondary compounds identified in a percentage of 80–90% of the samples one can find:

Dimethyl sulfide, acetone, 3-hydroxy-2-butanone, linalool oxide, acetic acid, 2-furancarboxaldehyde, benzaldehyde, linalool L, ho-trienol, 4-ketoisophorone, epoxylinalool, benzenemethanol for Zone 1;

Dimethyl sulfide, acetone, 3-hydroxy-2-butanone, 2-methyl-2-buten-1-ol, nonanal, linalool, 2-furancarboxaldehyde, 5-methyl-2-furancarboxaldehyde, butanoic acid, epoxy-linalool, 3-hydroxy-2-butanone, methylpentanoic acid, benzenethanol for Zone 2;

Dimethyl sulfide, ethanol, isoamyl alcohol, 2-methyl-2-buten-1-ol, *cis*-3-hexen-1-ol, linalool oxide, acetic acid, 2-furancarboxaldehyde, benzaldehyde, propanoic acid, hotrienol, butyrolactone, benzeneacetaldehyde, phenol for Zone 3.

Thus, once the qualitative analysis was completed, the volatile profile of the Romanian acacia honey was outlined.

The results revealed that there is a remarkable influence of the production zone on the volatile profile of the honey samples. Of all the identified compounds, only 56 are common to all the 50 samples. The chemotype changes from one region to another, and the difference resides in the 23 compounds found only in certain regions. The compounds found only in Zone 1 are: 2-butanol, 5-methyl-2-hexanone, 2-heptanone, octanal, 2,2-dymethyl propanoic acid, naphthalene, nonanoic and octanoic acids. The origin of these compounds can be either vegetal (nectar and poliniferous plants) or animal, products secreted by the bee. For example, octanol is found in *Houttuynia cordata* and in *Lavandula angustifolia* [56]. The 2-heptanone compound was identified in the secretion of bees by Shearer [57], but it is also found in *Artemisia dracunculus* and *Larrea tridentate* [58,59]. In regard to Zone 2, borneol and HMF compounds have been identified. Borneol is a monoterpenic compound found in medicinal plants [60]. Borneol is used as an additive in cosmetic products due to its analgesic and antibacterial effects [61]. The presence of the HMF compound is a hint towards honey freshness [62]. In Zone 3, pentanoic acid was found. Also, it was found that Zone 1 shows a volatile profile similar to Zone 3, a relevant example being the existing common compounds, *beta*-damascenone, hotrienol, benzyl nitrile, 2,5-furancarboxaldehyde. Out of these compounds, *beta*-damascenone was found in *Ipomoea pes caprae* with an antispastic effect [63]. Additionally, hotrienol was found to be a component of many honey varieties, and of essential oils of many plants. This compound was found to be specific to citrus, lavender, and mint honey [64,65,66].

By using the peak area, it is possible to determine the percentage of each compound, compared to the total surface of the areas.

The calibration and concentration determination methods for volatile compounds are:

Gauging of the calibration curve method (area normalization);

Internal standard method [67].

For a better interpretation of the results, the first method was used, the concentration of each compound being calculated according to the following expression:

X% = (A_x_/∑A_i_), (3)

where: A_x_: compound surface, ∑A_i_: the sum of all identified compound surfaces.

Table 8 shows the semi-quantification results for each product, as well as product chemical classification. The volatile compounds identified in the acacia honey samples stemming from the three geographical areas are classified into 10 chemical subclasses: Sulphur compounds, ketones, esters, aldehydes, alcohols, nitrate compounds, aliphatic hydrocarbons, carboxylic acids, aromatic acids and ethers. From the collected data, we can see that the honey samples harvested from Zone 1 (Transylvania) are the richest in volatile compounds, as all of these compound classes are present in the samples. Keeping in mind that the number of compounds was so high, only the majorities were discussed.

Regarding the presence of acetic acid, this compound was found in samples from all three zones, but in different quantities. Acetic and butyric acids could be produced by bee metabolism [68]. This is the main compound of the honey samples, and its percentages vary between 12.26% for Zone 3, 16.64% for Zone 1 and 24.73% for Zone 2. The previous studies show that the percentage of this compound can vary between 17.32% and 36.15% for different Chilean honey types [19], while having a percentage of 0.01% in Spanish honey [64]. We must mention that the two studies have used different extraction methods, ultrasound extraction and thermal desorption. Kadar et al. [21] have found that Romanian acacia honey has linalool oxide as the main compound, without having identified any acetic acid.

Ethanol is another important constituent of honey, all honey samples have shown a high ethanol content, especially those from Zone 2 and Zone 3, as their content reached 17.91% and 13.07%, respectively. In the case of Transylvanian honey (Zone 1), there was a relatively low ethanol content found, of 4.11%. Bastos and Alves [68] issue the hypothesis that the high ethanol content is due to the presence of ferments. Ethanol was found to be a marker for lavender honey [65]. Furthermore, ethanol was found in honeys of different geographical and botanical origins [8,9,22,64].

The volatile profile found depends on the extraction method. Therefore, the fiber used shows a great affinity for compounds with low molecular mass, like ethanol or acetic acid [69], which justifies the significant presence of all these compounds in the honey samples, from a quantitative point of view.

Another major compound was 2-propanone, present in a high concentration in Zone 3 (20.60%), as opposed to its presence in honey stemming from Zone 1 and Zone 2, where its values were 2.83% and 5.11%, respectively. Transylvanian honeys (Zone 1) have shown the lowest 2-propanone or acetone content, these differences being the result of the discrepancy between geographical, climatic and especially floral conditions. In a recent study, acetone concentration in Chilean honey was determined, according to geographical area. In the eastern part, the zone percentage was between 1.57–17.15%, in the central area, 7.8–9.63%, and in the western zone, 5.00–13.13% [19]. Acetone is considered a marker for pine honey (*Albies* spp.) [68]. Additionally, acetone was found to be a major compound in acacia and rosemary monofloral honey [65]. Linalool oxide and 2–furancarboxaldehyde are usually found in the honey samples collected from Zone 2 (8.61% and 9.73). Linalool oxide is a compound of floral origin (Pherobase) and is considered to be a secondary product of linalool. Jerkovic et al. [70] determined a 2.23% concentration of this product in acacia honey, whereas in chestnut honey, the concentration was of 0.40%.

Furfural is a compound derived from furan, which is considered to be an indicator of the thermal and storage processes [64]. On the other hand, furfural was identified as being a relevant compound in unifloral lime tree, lavender and acacia honey [65]. Furfural is present in all regions with an area percentage of 9.73% for Zone 2, 7.26% for Zone 1 and 5.24% for Zone 3. Radovic et al. [71] established furfural as being a marker for rape honey. These volatile compounds, furfural and 5-methyl-furfural were identified in fresh citrus honey. Moreover, the evolution of the concentration of these compounds was observed and it intensified during storage and temperature increase from 10 to 40 °C [64]. The mild heating of the samples during the SPME analysis recommended for the improvement of the extraction result and the reduction of the balance time, may be responsible for some of these compounds [72].

We can also observe a higher benzaldehyde content, especially in Zone 1 honey (7.23%), as compared to the one in Zone 2 (3.89%) and Zone 3 (4.68%). Benzaldehyde was identified as being a relevant compound in lavender, acacia and rosemary honey [65,72]. Yang et al. [73] identified benzaldehyde as being a volatile marker in Corsican chestnut honey with a 10.8% concentration. Furthermore, we can also note the presence of benzeneethanol in significant amounts in the case of the honey samples from Zone 2 (3.58%), Zone 1 (2.78%) and Zone 3 (2.37%). Benzenethanol and benzoic acid were identified as rape honey markers [74].

Alcohols constitute an important part of Romanian honey samples reaching values of 28.35% in Zone 3, 23.87% in Zone 2, 19.13% in Zone 1. The alcohols present in all the zones were ethanol, 2-propanol, 2-methyl-3-butene-2-ol, isoamyl alcohol, 3-methyl-3-butene-1-ol, 2-methyl-2-butene-1-ol, *cis*-3-hexen-1-ol, 2-ethyl-1-hexanol, linalool, hotrienol, *alfa*-terpienol, benzeneethanol, benzenemethanol, phenol. Among the identified alcohols, ethanol, benzeneethanol, 3-methyl-3-butene-1-ol proved to have the highest area percentage of all the analyzed samples (Table 8).

Alcohols represent important markers of honey; alcohols, such as 3-methyl-3-butene-1-ol and 2-methyl-2-butene-1-ol were described as adding a fresh aroma, their presence in honey being associated with different floral origins [64]. Ethanol, 2-methyl-propanol and 3-methyl-3-butene-1-ol were described as being very important compounds in monofloral lavender honey [65]. The identification of 3-methyl-3-butene-1-ol was also associated with monofloral rosemary honey [75].

As far as aldehydes are concerned, it can be observed that the honey samples from Zone 1 present the highest concentration, 27.63%, followed by the ones from Zone 2 with a 22.34% concentration. The samples in Zone 3 have a relative aldehyde concentration of 13.43%. The aldehydes identified in the samples from all the three production areas are: 3-methyl-butanal, heptanal, 3-methyl-2-butanal, nonanal, furfural, decanal, benzaldehyde, lilac aldehyde, 5-methyl-2-furancarboxaldehyde, acetaldehyde and 3-phenylfenil-propenal. Plutowska et al. [76] have established hexanal as a specific marker for the acacia honey produced in Poland, while methyl butanol and lilac aldehyde proved to be a specific marker for the Polish honeydew honey. The following substances from the aldehyde group were also identified in the composition of these honeys: hexanal, nonanal, decanal, acetaldehyde and lilac aldehydes.

Ketones, sulfur compounds, esters and aliphatic hydrocarbons were also identified in small amounts, but none of them could be considered markers for acacia honey.

As other studies presented the idea that minor compounds also influence honey flavor, the table was organized in order to summarize the specific aromas of all the identified volatile compounds. Thus, it can be observed that the honey aroma is very complex and depends on the specific flora of each production zone, but also on the compounds secreted by the bees or on the transformations that occur during storage. 

## 4. Conclusions

In accordance with the motivation and the objectives proposed, the quality indices and the biomarkers specific to acacia honey were established. However, pure acacia honey represents only a low percentage of the total honey production, as the majority of honeys have a varied composition of nectar or honeydew, being thus considered polyfloral honeys. Therefore, distinguishing monofloral honeys from polyfloral honeys is a significant challenge that involves assessing the botanical origin of honey and its correct labelling. Volatile compounds can be useful in classifying acacia honeys, but they are insufficient in order to distinguish between the samples produced in different areas. As such, the use of multiple volatile compounds is recommended, which will constitute the volatile fingerprint of a certain area, so that the results are less susceptible to the variation of individual components.

Many consumers seek for products with special aromas, specific to production zones. In conclusion, consumer affinity for the special aroma of the acacia honey, which have an important nutritional value, being recommended in small amounts even for diabetes patients [77], is legitimate and this study aims at scientifically explaining the fact that honey flavor is influenced by its origin.

## Figures and Tables

**Table 1:** Selective physico-chemical parameter values for investigated honey samples (water content (%) and water activity (a_w_)).

Zone 1			Zone 2			Zone 3		
Sample Code	Obtained Values		Sample Code	Obtained Values		Sample Code	Obtained Values	
	Water Content (%)	Water Activity (a_w_)		Water Content (%)	Water Activity (a_w_)		Water Content (%)	Water Activity (a_w_)
S1	16.8 ± 0.1	0.599 ± 0.000	S4	16.1 ± 0.2	0.568 ± 0.001	S5	17.3 ± 0.1	0.565 ± 0.001
S2	15.5 ± 0.1	0.573 ± 0.006	S6	17.4 ± 0.0	0.578 ± 0.001	S7	15.8 ± 0.2	0.593 ± 0.001
S3	16.9 ± 0.0	0.607 ± 0.001				S11	16.4 ± 0.0	0.578 ± 0.001
S8	16.0 ± 0.2	0.578 ± 0.001	S14	17.3 ± 0.1	0.594 ± 0.001	S21	16.8 ± 0.2	0.686 ± 0.004
S9	16.3 ± 0.1	0.602 ± 0.003				S29	17.2 ± 0.1	0.610 ± 0.001
S10	15.4 ± 0.0	0.575 ± 0.001	S19	17.8 ± 0.3	0.621 ± 0.001	S30	15.5 ± 0.2	0.574 ± 0.001
S12	15.7 ± 0.3	0.558 ± 0.003				S31	16.1 ± 0.1	0.615 ± 0.001
S13	17.5 ± 0.0	0.603 ± 0.001	S25	20.4 ± 0.1	0.690 ± 0.004	S32	16.5 ± 0.1	0.599 ± 0.000
S15	21.3 ± 0.2	0.600 ± 0.000				S33	16.9 ± 0.1	0.601 ± 0.000
S16	15.7 ± 0.1	0.562 ± 0.001	S27	17.0 ± 0.0	0.602 ± 0.000	S34	20.4 ± 0.1	0.656 ± 0.001
S17	18.0 ± 0.2	0.610 ± 0.003				S39	15.4 ± 0.1	0.555 ± 0.003
S18	17.1 ± 0.1	0.621 ± 0.000	S35	16.7 ± 0.2	0.598 ± 0.001	S40	18.9 ± 0.2	0.580 ± 0.001
S20	18.0 ± 0.1	0.616 ± 0.001						
S22	15.7 ± 0.1	0.559 ± 0.001	S36	16.8 ± 0.2	0.595 ± 0.001	S41	16.9 ± 0.3	0.574 ± 0.001
S23	16.6 ± 0.1	0.591 ± 0.000						
S24	16.7 ± 0.1	0.597 ± 0.001	S37	18.7 ± 0.1	0.620 ± 0.001	S42	16.9 ± 0.3	0.573 ± 0.000
S28	16.7 ± 0.1	0.592 ± 0.001	S38	16.7 ± 0.1	0.580 ± 0.002	S52	14.8 ± 0.2	0.537 ± 0.003
S48	17.1 ± 0.1	0.578 ± 0.001						
S51	15.0 ± 0.2	0.540 ± 0.001	S50	18.0 ± 0.2	0.596 ± 0.000	S55	17.2 ± 0.2	0.587 ± 0.000
S53	16.8 ± 0.1	0.570 ± 0.000						
S58	16.1 ± 0.0	0.552 ± 0.00	S57	16.1 ± 0.1	0.558 ± 0.001	S56	18.8 ± 0.1	0.611 ± 0.001
Average	16.705 ± 0.1	0.585 ± 0.001	Average	17.416 ± 0.13	0.600 ± 0.001	Average	17.088 ± 0.15	0.591 ± 0.01

Every value is a mean of three determinations (*n* = 3) ± standard deviations.

**Table 2:** The color parameter values of acacia honey samples (L*, a*, b*) (*n* = 50).

Zone 1				Zone 2				Zone 3			
Sample Code	Obtained Values			Sample Code	Obtained Values			Sample Code	Obtained Values		
	L*	a*	b*		L*	a*	b*		L*	a*	b*
S1	60.62 ± 0.02	−0.76 ± 0.01	13.02 ± 0.01	S4	54.11 ± 0.08	2.03 ± 0.02	25.99 ± 0.06	S5	60.48 ± 0.13	−0.39 ± 0.01	10.98 ± 0.03
S2	59.42 ± 0.03	−0.69 ± 0.02	13.06 ± 0.02	S6	50.19 ± 021	0.39 ± 0.02	19.60 ± 0.03	S7	56.20 ± 0.12	0.22 ± 0.02	24.91 ± 0.00
S3	51.28 ± 0.09	1.98 ± 0.02	26.07 ± 0.03					S11	59.60 ± 0.04	−0.62 ± 0.01	12.81 ± 0.04
S8	59.62 ± 0.09	−0.54 ± 0.04	15.32 ± 0.05	S14	56.94 ± 0.26	0.98 ± 0.03	21.53 ± 0.06	S21	56.96 ± 0.10	0.58 ± 0.03	21.17 ± 0.15
S9	60.24 ± 0.08	−0.42 ± 0.01	13.34 ± 0.03					S29	60.10 ± 0.07	−0.76 ± 0.01	13.68 ± 0.09
S10	58.16 ± 0.06	−0.37 ± 0.02	19.27 ± 0.09	S19	57.76 ± 0.03	0.50 ± 0.03	17.23 ± 0.05	S30	59.17 ± 0.14	0.29 ± 0.02	17.45 ± 0.08
S12	59.52 ± 0.05	−0.11 ± 0.03	15.85 ± 0.01					S31	57.03 ± 0.19	0.44 ± 0.04	20.23 ± 0.39
S13	58.96 ± 0.22	0.08 ± 0.02	16.79 ± 0.04	S25	57.56 ± 0.11	0.52 ± 0.01	17.73 ± 0.09	S32	55.73 ± 0.37	1.18 ± 0.01	22.19 ± 0.28
S15	54.48 ± 0.18	2.31 ± 0.01	28.22 ± 0.05					S33	55.88 ± 0.18	0.98 ± 0.02	21.84 ± 0.14
S16	61.87 ± 0.19	−0.36 ± 0.03	9.47 ± 0.11	S27	57.82 ± 0.28	0.57 ± 0.03	20.82 ± 013	S34	55.42 ± 0.16	1.20 ± 0.02	23.23 ± 0.07
S17	60.51 ± 0.17	−0.23 ± 0.03	13.07 ± 0.10					S39	59.84 ± 0.11	−0.40 ± 0.00	18.19 ± 0.09
S18	50.92 ± 0.19	5.80 ± 0.06	30.41 ± 0.59	S35	59.69 ± 0.11	0.08 ± 0.03	13.08 ± 0.03	S40	60.46 ± 0.08	−0.60 ± 0.01	12.05 ± 0.09
S20	60.98 ± 0.07	0.68 ± 0.03	11.20 ± 0.11								
S22	59.68 ± 0.20	0.23 ± 0.02	16.56 ± 0.08	S36	60.98 ± 0.40	−0.29 ± 0.00	11.39 ± 0.18	S41	62.60 ± 0.13	−0.81 ± 0.02	7.03 ± 0.03
S23	60.54 ± 0.04	−0.37 ± 0.01	11.75 ± 0.02								
S24	60.12 ± 0.02	−0.11 ± 0.02	14.76 ± 0.03	S37	57.48 ± 0.07	0.62 ± 0.03	19.82 ± 0.18	S42	61.34 ± 0.10	−0.70 ± 0.02	10.38 ± 0.06
S28	59.28 ± 0.16	0.02 ± 0.02	16.73 ± 0.14	S38	60.97 ± 0.38	−0.62 ± 0.01	12.07 ± 0.22	S52	62.70 ± 0.18	−0.99 ± 0.03	9.87 ± 0.07
S48	64.00 ± 0.12	−0.66 ± 0.01	6.23 ± 0.04								
S51	59.88 ± 0.38	−0.71 ± 0.02	20.67 ± 0.32	S50	62.59 ± 0.03	−1.05 ± 0.03	7.21 ± 0.03	S55	61.05 ± 0.12	−0.73 ± 0.01	11.25 ± 0.08
S53	61.11 ± 0.06	−0.68 ± 0.01	13.60 ± 0.10								
S58	61.96 ± 0.43	−1.28 ± 0.04	11.37 ± 0.05	S57	62.03 ± 0.35	−0.82 ± 0.02	8.66 ± 0.04	S56	59.90 ± 0.00	−0.27 ± 0.03	14.07 ± 0.10
Average	59.31 ± 0.14	0.12 ± 0.02	15.16 ± 0.10	Average	58.18 ± 0.18	0.24 ± 0.02	16.26 ± 0.09	Average	59.06 ± 0.13	−0.08 ± 0.02	15.96 ± 0.11

L* - degree of brightness (luminosity); a*, b* - color tonality.

**Table 3:** Analysis of variance for water content, water activity and color (L, a*, b*).

Parameter/Zone		Analysis of Variance Parameters	
**Water Content (%)**			
Groups	Count	Average	Variance
Zone 1	21	16.70476	1.789726
Zone 2	12	17.41667	1.463333
Zone 3	17	17.08824	2.327353
ANOVA	F	*p*-value	F crit
	1.06635	0.352444	3.195056
**Water activity (a_w_)**			
Groups	Count	Average	Variance
Zone 1	21	0.585333	0.000510
Zone 2	12	0.6000	0.001154
Zone 3	17	0.591412	0.001423
ANOVA	F	p-value	F crit
	0.848591	0.43447	3.195056
Groups	Count	Average	Variance
Zone 1	21	59.31476	9.517026
Zone 2	12	58.17667	12.29979
Zone 3	17	59.06235	5.631544
ANOVA	F	p-value	F crit
	0.575627	0.566267	3.195056
a*			
Groups	Count	Average	Variance
Zone 1	21	0.118095	2.189636
Zone 2	12	0.2425	0.72793
Zone 3	17	−0.08118	0.544336
ANOVA	F	p-value	F crit
	0.306413	0.737542	3.195056
b*			
Groups	Count	Average	Variance
Zone 1	21	15.16219	40.39225
Zone 2	12	16.26417	32.77234
Zone 3	17	15.96059	30.49048
ANOVA	F	p-value	F crit
	0.156758	0.855356	3.195056

**Table 4:** Pearson correlation matrix of physico-chemical parameters for Zone 1.

Parameters	Water content	Fructose	Glucose	Sucrose	Turanose	Maltose	Isomaltose	Erlose	Water activity	Color L*	Color a*	Color b*
**Water content**	1.000											
**Fructose**	−0.133	1.000										
**Glucose**	0.071	−0.476	1.000									
**Sucrose**	0.277	0.136	−0.539	1.000								
**Turanose**	−0.034	0.425	−0.371	−0.078	1.000							
**Maltose**	−0.209	0.419	−0.190	−0.045	0.721	1.000						
**Isomaltose**	0.098	−0.325	0.294	−0.527	0.333	0.110	1.000					
**Erlose**	0.167	0.374	−0.592	0.790	0.209	0.281	−0.441	1.000				
**Water activity**	0.581	−0.321	0.624	−0.359	−0.120	−0.139	0.471	−0.393	1.000			
Color L*	−0.009	0.378	−0.518	0.161	0.270	0.208	−0.185	0.369	−0.447	1.000		
Color a*	0.167	−0.612	0.708	−0.262	−0.385	−0.511	0.292	−0.527	0.549	−0.841	1.000	
Color b*	0.027	−0.411	0.435	−0.021	−0.379	−0.350	0.020	−0.266	0.288	−0.818	0.723	1.000

L^*^- degree of brightness (luminosity); a^*^, b^*^- color tonality; Blue: significant for the 0.01 level; Yellow: significant of the 0.05 level.

**Table 5:** Pearson correlation matrix of physico-chemical parameters for Zone 2.

Parameters	Water content	Fructose	Glucose	Sucrose	Turanose	Maltose	Isomaltose	Erlose	Water activity	Color L*	Color a*	Color b*
**Water content**	1.000											
**Fructose**	−0.271	1.000										
**Glucose**	0.343	−0.590	1.000									
**Sucrose**	−0.457	−0.194	0.332	1.000								
**Turanose**	−0.555	0.602	−0.584	−0.139	1.000							
**Maltose**	−0.601	0.211	0.178	0.372	0.589	1.000						
**Isomaltose**	−0.117	−0.107	0.078	−0.264	0.577	0.519	1.000					
**Erlose**	−0.768	0.133	−0.270	0.519	0.640	0.695	0.376	1.000				
**Water activity**	0.921	−0.321	0.500	−0.367	−0.676	−0.522	−0.182	−0.762	1.000			
Color L*	−0.095	0.564	−0.160	−0.011	0.286	0.092	−0.163	−0.083	−0.014	1.000		
Color a*	0.048	−0.263	0.403	0.157	−0.220	0.292	0.153	0.177	0.137	−0.728	1.000	
Color b*	0.092	−0.336	0.382	0.059	−0.175	0.299	0.264	0.175	0.134	−0.803	0.962	1.000

L^*^- degree of brightness (luminosity); a^*^, b^*^- color tonality; Blue: significant for the 0.01 level; Yellow: significant of the 0.05 level.

**Table 6:** Pearson correlation matrix of physico-chemical parameters for Zone 3.

Parameters	Water content	Fructose	Glucose	Sucrose	Turanose	Maltose	Isomaltose	Erlose	Water activity	Color L*	Color a*	Color b*
**Water content**	1.000											
**Fructose**	−0.097	1.000										
**Glucose**	−0.013	−0.891	1.000									
**Sucrose**	−0.482	0.106	0.049	1.000								
**Turanose**	−0.414	0.655	−0.693	0.450	1.000							
**Maltose**	−0.367	0.664	−0.611	0.485	0.947	1.000						
**Isomaltose**	−0.359	0.273	−0.202	0.200	0.669	0.745	1.000					
**Erlose**	−0.264	0.684	−0.666	0.513	0.908	0.935	0.566	1.000				
**Water activity**	0.744	−0.428	0.337	−0.750	−0.745	−0.723	−0.411	−0.719	1.000			
Color L*	−0.334	0.157	−0.165	0.667	0.565	0.572	0.243	0.733	−0.716	1.000		
Color a*	0.363	−0.077	0.022	−0.796	−0.473	−0.464	−0.125	−0.619	0.708	−0.944	1.000	
Color b*	0.177	−0.299	0.255	−0.576	−0.548	−0.580	−0.224	−0.749	0.624	−0.949	0.876	1.000

L^*^- degree of brightness (luminosity); a^*^, b^*^- color tonality; Blue: significant for the 0.01 level; Yellow: significant of the 0.05 level.

**Table 7:** Retention index from literature (Ri^a^) and calculated retention index (RI^b^) of volatile compounds in honey samples of the three investigated zones (Z1–Z3).

Rank	Volatile Compound (Usual and/or IUPAC Name)	RI^a^	Z1	Z2	Z3
			RI^b^	RI^b^	RI^b^
1	dimethyl sulfide/methylsulfanylmethane	734			
2	acetone/2-propanone	810	807	806	805
3	ethyl acetate	872			862
4	3-methylbutanal/Isovaleraldehyde	912	906	906	904
5	isopropyl alcohol/2-propanol	917	915	919	913
6	ethanol	928	923	923	921
7	2,2,4,6,6-pentamethylheptane				945
8	2,3-butanedione/diacetyl	977	969	967	968
9	2-methylpropanenitrile/isobutyronitrile		985		976
10	*alfa*-pinene/2,6,6-trimethylbicyclo[3.1.1]hept-2-ene		999	999	1000
11	2-butanol/sec-butanol	1022	1010		
12	3-methylpentanal/3-methylvaleraldehyde		1016	1010	1011
13	2-methyl-3-buten-2-ol/dimethylvinylcarbinol	1036	1041	1024	1013
14	3,6-dimethyldecane		1051		1054
15	hexanal	1067	1061		
16	2-methyl-2-butenal			1069	
17	2-methylpropan-1-ol/isobutyl alcohol/isobutanol	1085			1077
18	3-pentanol	1110	1090	1084	1086
19	*delta*-3-carene/3,7,7-trimethylbicyclo[4.1.0]hept-3-ene	1148	1122	1127	1104
20	5-methyl-2-hexanone		1129		
21	heptanal	1184	1162	1155	1164
22	2-heptanone	1160	1169		
23	limonene	1198	1171	1169	
24	3-methyl-2-butenal		1174	1173	1174
25	dodecane	1200	1181	1181	1180
26	isoamyl alcohol/3-methylbutan-1-ol	1230	1183	1184	1183
27	3-methyl-3-buten-1-ol	1240	1223	1223	1221
28	3-hydroxy-2-butanone/acetoin/3-hydroxy-2-butanone	1272	1269	1254	1255
29	octanal	1278	1269		
30	2-methyl-2-buten-1-ol	1315	1294	1294	1293
31	6-methyl-5-hepten-2-one	1329	1312	1350	1304
32	3-hydroxy-3-methylbutanoic acid		1347	1350	1345
33	*cis*-3-hexene-1-ol	1351	1360	1362	1358
34	nonanal	1382	1371	1377	1369
35	linalool oxide/6-ethenyl-2,2,6-trimethyloxan-3-ol	1423	1415	1412	1412
36	acetic acid	1432	1434	1432	1430
37	2-furancarboxaldehyde/furfural	1434	1441	1440	1435
38	*trans*-linalool oxide	1451	1445	1442	1443
39	2-ethyl-1-hexanol		1472	1477	1473
40	decanal	1485	1481	1480	1478
41	2-acetylfuran	1491	1482	1482	1480
42	benzaldehyde	1502	1495	1494	1493
43	lilac aldehyde B		1519	1524	1516
44	propanoic acid/propionic acid	1523	1526	1528	1520
45	linalool L	1544	1532	1527	1528
46	lilac aldehyde A		1545	1544	1543
47	5-methyl-2-furancarboxaldehyde	1563	1553	1551	1551
48	2-methylpropanoic acid	1584	1555	1558	1553
49	2,2-dimethylpropanoic acid/pivalic acid		1585		
50	ho-trienol	1586	1594		1592
51	butyrolactone		1600	1603	1596
52	butanoic Acid	1628	1615	1617	1612
53	benzenacetaldehyde/acetaldehyde	1646	1622	1622	1618
54	5-ethenyldihydro-5-methyl-2(3H)-Furanone		1647	1644	1641
55	2-methylbutanoic acid/DL-2-methylbutyric acid	1667	1661	1662	1662
56	4-ketoisophorone/4-oxoisophorone		1672		1669
57	*alfa*-terpineol/(S)-2-(4-Methyl-3-cyclohexenyl)-2-propanol	1688	1675	1677	1680
58	3-pyridinecarboxaldehyde/nicotinic aldehyde		1676		1682
59	borneol	1698		1684	
60	naphtalene	1718	1718		
61	epoxylinalool		1728	1732	1722
62	pentanoic acid/valeric acid				1729
63	3-methylpentanoic acid	1780	1783	1784	1786
64	*beta*-damascenone	1790	1804		1801
65	hexanoic acid	1814	1836	1837	1835
66	*para*-cymen-8-ol/2-(4-methylphenyl)propan-2-ol		1847	1838	1838
67	*trans*-geranylacetone/(5E)-6,10-dimethylundeca-5,9-dien-2-one		1841	1846	1837
68	benzenemethanol/phenylmethanol	1844	1862	1860	1847
69	benzeneethanol/phenethyl alcohol	1878	1895	1893	1891
70	benzyl nitrile/2-Phenylacetonitrile				
71	sabinene/4-methylidene-1-propan-2-ylbicyclo[3.1.0]hexane				
72	2,5-furandicarboxaldehyde/furan-2,5-dicarbaldehyde				
73	phenol	1932			
74	octanoic acid/caprylic acid	2083			
75	nonanoic acid/pelargonic acid	2110			
76	3-phenyl-2-propenal/cinnamaldehyde				
77	2-methoxy-4-vinylphenol				
78	benzoic acid				
79	HMF/5-hydroxymethil-2-Furfural				

**Table 8:** Relative area of volatile organic compounds (VOC) of honey samples from the three investigated zones (Z1 – Z3).

Rank	Compound	Zone 1 (%)	Zone 2 (%)	Zone 3 (%)	Class	%COV Z1	%COV Z2	%COV Z3
1	dimethyl sulfide/methylsulfanylmethane	1.578	2.867	2.171	Sulphur compounds	1.578	2.867	2.171
2	acetone/2-propanone	2.830	5.119	20.609	Ketone	7.463	7.433	23.058
8	2,3-butanedione/diacetyl	1.387	1.420	0.765				
20	5-methyl-2-hexanone	0.066	0.000	0.000				
22	2-heptanone	0.542	0.000	0.000				
28	3-hydroxy-2-butanone/acetoin	0.708	0.646	0.853				
31	6-methyl-5-hepten-2-one	0.188	0.171	0.125				
56	4-ketoisophorone/4-oxoisophorone	0.963	0.000	0.510				
64	*beta*-demascenone	0.209	0.000	0.072				
67	*trans*-geranylacetone	0.570	0.077	0.124				
3	ethyl acetate	0.000	0.000	2.720	Esters	0.000	0.000	2.720
4	3-methyl butanal/isovaleraldehyde	1.753	0.538	0.002	Aldehyde	27.639	22.344	13.439
15	hexanal	0.178	0.000	0.000				
17	2-methyl-2-butenal	0.447	0.370	0.000				
21	heptanal	0.074	0.020	0.004				
24	3-methyl-2-butenal	1.514	0.522	0.165				
29	octanal	0.020	0.000	0.000				
34	nonanal	1.382	1.183	0.575				
37	2-Furancarboxaldehyde / furfural	7.262	9.737	5.241				
41	decanal	0.648	4.464	0.070				
42	benzaldehyde	7.237	3.890	4.686				
43	lilac aldehyde B	1.898	0.177	0.141				
46	lilac aldehyde A	2.430	0.017	0.046				
47	5-methyl-2-furancarboxaldehyde	0.321	0.516	0.156				
53	benzenacetaldehyde/acetaldehyde	1.088	0.809	1.945				
59	3-pyridinecarboxaldehyde/nicotinic aldehyde	0.706	0.000	0.095				
72	2,5-furandicarboxaldehyde/furan-2,5-dicarbaldehyde	0.670	0.000	0.292				
76	3-phenyl-2-propenal/cinnamaldehyde	0.011	0.097	0.021				
79	HMF/5-hydroxymethyl-2-Furfural	0.000	0.004	0.000				
5	isopropyl alcohol/2-propanol	0.864	0.003	0.265	Alcohols	19.130	23.876	28.359
6	ethanol	4.144	13.077	17.910				
11	2-butanol/sec-butanol	0.007	0.000	0.000				
13	2-methyl-3-buten-2-ol/dimethylvinylcarbinol	0.442	0.493	0.122				
16	2-methyl-1-propanol/isobutanol	0.000	0.000	0.101				
18	3-pentanol	0.007	0.096	0.011				
25	isoamyl alcohol	0.405	0.510	1.171				
27	3-methyl-3-buten-1-ol	2.090	1.335	0.565				
30	2-methyl-2-buten-1-ol	0.716	0.713	0.208				
33	*cis*-3-hexene-1-ol	0.078	0.193	0.145				
39	2-ethyl-1-hexanol	0.174	0.028	0.098				
45	linalool L	3.101	1.418	1.930				
50	ho-trienol	2.636	0.000	1.340				
57	*alfa*-terpineol	0.158	0.344	0.039				
58	borneol	0.000	0.005	0.000				
61	epoxylinalol	0.532	0.584	0.292				
66	*para*-cymen-8-ol/2-(4-methylphenyl)propan-2-ol	0.007	0.006	0.003				
68	benzenemethanol	0.850	1.279	1.623				
69	benzeneethanol/phenethyl alcohol	2.780	3.683	2.378				
73	Phenol	0.139	0.109	0.158				
7	2,2,4,6,6-pentamethylheptane	0.000	0.000	0.021	Aliphatic hydrocarbons	0.937	0.263	0.608
10	*alfa*-pinene/4,7,7-trimethylbicyclo[3.1.1]hept-3-ene	0.026	0.026	0.012				
12	3-methylpentanal/3-methylvaleraldehyde	0.608	0.155	0.498				
14	3,6 dimethyldecane	0.156	0.000	0.024				
19	*delta*-3-carene/3,7,7-trimethylbicyclo[4.1.0]hept-3-ene	0.035	0.006	0.018				
23	limonene	0.056	0.073	0.016				
26	dodecane	0.003	0.003	0.019				
71	sabinene/4-methylidene-1-propan-2-bicyclo[3.1.0]hexane	0.053	0.000	0.000				
9	2-methylpropanenitrile	0.326	0.000	0.045	Nitrogen compounds	0.916	0.000	1.610
70	benzylnitrile	0.590	0.000	1.565	
32	3-hydroxy-3-methylbutanoic acid	0.025	0.074	0.070	Carboxylic acids	32.225	30.369	21.723
36	acetic acid	16.641	24.733	12.269				
44	propanoic acid/propionic acid	0.862	0.697	0.687				
48	2-methylpropanoic acid	1.335	0.203	1.385				
49	2,2-dimethyl propanoic acid/Pivalic acid	0.052	0.000	0.000				
52	butanoic acid	1.646	0.750	0.644				
55	2-methylbutanoic acid/DL-2-methylbutyric Acid	5.094	1.771	1.219				
62	pentanoic acid/valeric acid	0.000	0.000	0.108				
63	3-methylpentanoic acid	5.336	1.629	4.497				
65	hexanoic acid	0.661	0.507	0.265				
74	octanoic acid	0.172	0.000	0.000				
75	nonanoic acid	0.092	0.000	0.000				
78	benzoic acid	0.309	0.005	0.579				
35	linalool oxide/6-ethenyl-2,2,6-trimethyloxan-3-ol	6.883	8.618	4.188	Aromatic acids	9.698	12.790	6.211
38	*trans*-linalool oxide	1.564	2.162	1.115				
40	2-acetylfuran	0.121	0.435	0.267				
51	butyrolactone	0.224	0.174	0.274				
54	5-ethenyldihydro-5-methyl-2(3H)-furanone	0.888	1.401	0.367				
60	naphtalene	0.018	0.000	0.000				
77	2-methoxy-4-vinylphenol	0.085	0.057	0.103	Ethers	0.085	0.057	0.103

## Supplementary Materials

The following are available online at https://www.mdpi.com/2304-8158/8/10/445/s1, Figure S1: *CIELAB* color space (www.printroot.com); Figure S2: Descriptive statistics for water content of Romanian acacia honey and box plot graph for the three production zones, Figure S3: GC-MS chromatogram of VOC in sample S1.
