# Supplementary material for: Volatile Profile and Physico-Chemical Analysis of Acacia Honey for Geographical Origin and Nutritional Value Determination
Source: Foods. 2019 Sep 27;8(10):445. doi: 10.3390/foods8100445 (PMC6836064; doi:10.3390/foods8100445)
Supplement: Supplementary file 1 [file foods-08-00445-s001.zip › Figure S 1.doc]

Supplementary Figure 1. *CIELAB color space* (www.printroot.com)
