# Supplementary material for: Volatile Profile and Physico-Chemical Analysis of Acacia Honey for Geographical Origin and Nutritional Value Determination
Source: Foods. 2019 Sep 27;8(10):445. doi: 10.3390/foods8100445 (PMC6836064; doi:10.3390/foods8100445)
Supplement: Supplementary file 1 [file foods-08-00445-s001.zip › Figure S 2.doc]

Supplementary Figure 2. Descriptive statistics for water content of Romanian acacia honey and box plot graph for the three production zones
